# Oxytocin’s dynamic role across the lifespan

**DOI:** 10.1016/j.nbas.2021.100028

**Published:** 2022-01-25

**Authors:** Kristin Audunsdottir, Daniel S. Quintana

**Affiliations:** a Department of Psychology, University of Oslo, Oslo, Norway; b Norwegian Centre for Mental Disorders Research (NORMENT), University of Oslo, Oslo, Norway; c KG Jebsen Centre for Neurodevelopmental Disorders, University of Oslo, Oslo, Norway; d NevSom, Department of Rare Disorders, Oslo University Hospital, Oslo, Norway

**Keywords:** Oxytocin, Aging, Gene expression

## Abstract

Older adults have been neglected in biobehavioral oxytocin research. Emerging research indicates that oxytocin signaling activity fluctuates over the lifespan, which suggests that results from studies investigating youth and young adults cannot be easily generalized to older adults. The recruitment of a wider age range of research participants using a variety of research tools is required to uncover the role of the oxytocin signaling system over the lifespan and may reveal novel treatment target candidates in older adults, beyond social cognition and behavior.

## Introduction

Oxytocin is a neuropeptide primarily synthesized in the hypothalamus that helps coordinate a broad range of somatic processes [12]. Oxytocin was discovered over a century ago for its role in childbirth and lactation, but it has since received considerable research attention for its role in social cognition [14]. The recently proposed allostatic theory of oxytocin integrates oxytocin’s role in social and non-social cognition with its somatic effects, suggesting that oxytocin is an allostatic hormone that maintains stability throughout changing environments via its influence on perception, learning, prediction, and response [19].

Human oxytocin research in the biobehavioral sciences has primarily recruited youth and young adult samples, with only a small number of studies investigating health and cognition in older populations (e.g., [3], [6], [10], [18]). This is a remarkable situation considering that older adults are the fastest growing population in industrialized nations [25] and that increasing age is linked with declines in health and social cognition [17]. Each developmental stage is associated with unique priorities in terms of health and reproduction, so it is likely that oxytocin’s role in behavioral and somatic processes shifts throughout the lifespan to help manage these varied challenges [19]. Consequently, results from oxytocin studies with participants from a single developmental stage may not necessarily generalize across all developmental stages. Thus, there is currently a large knowledge gap on the role of oxytocin in cognition and somatic processes in older adults.

To help remedy this situation, a recent study by Valdes-Hernandez and colleagues [26] investigated the effects of chronic intranasal oxytocin administration in 31 older adults [mean age (SD) = 69 (6)] on brain mechanisms underlying animacy perception, which is the tendency to attribute motivation and meaning from living things to non-living or inanimate objects based on their motion. Animacy perception was assessed with the Heider-Simmel test [8], in which participants watch video clips of simple moving geometric shapes. The test includes a social condition, where the shapes’ movements suggest goal-directed behavior, and a non-social condition where movements seem random and non-goal-directed. Previous work using young adult samples found that intranasal oxytocin enhances anthropomorphizing of moving shapes that suggest a “social story”, but not for random motion [23], and an increased tendency to interpret social movement as “friendly social interactions” [7].

Using fMRI, Valdes-Hernandez and colleagues [26] did not observe any effects of oxytocin on brain activity in the social vs. random condition of the Heider-Simmel test. However, they did observe that the pre-to-post intervention change in the social-random difference in functional connectivity was higher in the oxytocin group compared to the placebo group in occipital, temporal, and parietal brain regions, as well as the superior temporal sulcus, a key structure in animacy perception. The authors interpret these findings to suggest that while chronic oxytocin administration does not modulate fMRI activation, it *does* modulate functional connectivity of brain regions involved in social perception in older adults. These observations are consistent with neuroplastic modification by chronic oxytocin administration, which adds to our understanding of oxytocin's effects on brain function and the therapeutic potential of chronic administration. But perhaps more importantly, this study also provides a greater insight into the role of the oxytocin system in older adults.

We currently lack a comprehensive understanding of how oxytocin signaling changes throughout the human lifespan, from the prenatal phase throughout late adulthood, and how these dynamics impact health, cognition, and behavior. As noted by Valdes-Hernandez and colleagues [26], one limitation of their study was that it did not include a comparison with a younger sample. While Horta and co-workers (2020) have put forward a compelling call to better understand the role of oxytocin across the lifespan via longitudinal and cross-sectional intranasal oxytocin studies across a range of age groups, other methods can be used to complement intranasal oxytocin investigations. Genetic approaches can be particularly informative, but this line of work generally focuses on how oxytocin receptor gene (*OXTR*) single nucleotide polymorphisms (SNPs) influence cognition and behavior over the lifespan. An alternative to the SNP approach is to evaluate how *OXTR* expression levels shift over the lifespan, which reflects changes in the distribution and activity of oxytocin binding [27]. As *OXTR* expression levels in the brain influence behavior [13], identifying *OXTR* expression dynamics in the brain across the lifespan and genes with similar spatiotemporal expression profiles can help identify critical lifespan periods for oxytocin signaling and the functional relevance of these fluctuations for both somatic and cognitive processes.

**Fig. 1:**
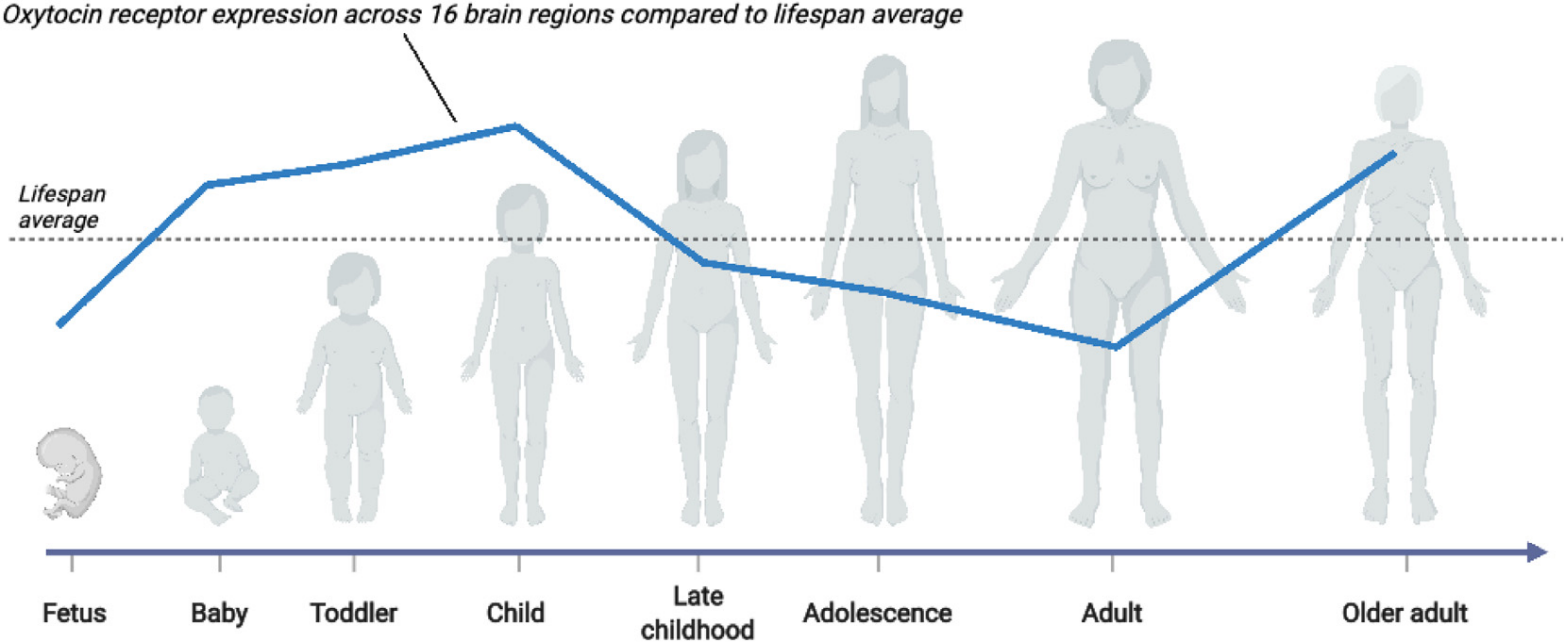
Oxytocin receptor expression in the brain across the lifespan**.** Oxytocin receptor gene expression is increased in both early childhood and older adulthood across the brain. This lifespan oxytocin receptor expression pattern was derived from Rokicki and colleagues (2021), who used genome-wide exon-level transcriptome data from 57 donors across 16 brain regions.

Recent cross-sectional research using human brain samples from 57 donors revealed a distinct *OXTR* expression pattern across the lifespan [22]. Specifically, *OXTR* expression was shown to first peak during childhood, which is a critical period for social learning. Following this lifespan period, *OXTR* expression across the brain reduced during early adulthood, then peaked again in late adulthood (Fig. 1). Increased oxytocin receptor expression in late adulthood was especially pronounced in the cerebellar cortex, which coordinates goal-directed movement and cognition [24]. We speculate that this late life increase in *OXTR* expression could be a compensatory mechanism to stave off cognitive declines due to age-related deterioration of cognitive circuitry in the brain, in support of oxytocin’s suggested role in maintaining allostasis [19]. Rokicki and colleagues [26] also reported that the genes with the strongest spatiotemporal correlation in the brain with the oxytocin receptor gene are associated with learning (*DRD2*), bone regeneration (*DIRAS3*), and energy homeostasis (*INHBB*), which points to a strong interplay between these genes and *OXTR*. Intriguingly, research has found that oxytocin administration in animals can reverse bone deterioration [2], which is especially relevant for older populations. Furthermore, individuals with autism spectrum disorder, a neurodevelopmental disorder characterized by social cognitive- and behavioral impairments, are also reportedly at increased risk for fracture and lower bone density compared to healthy controls [5]. Such results highlight how future research on oxytocin across the lifespan should not only focus on cognition, but also extend to somatic processes, such as bone density and energy regulation, and how these relate to cognition [19].

Despite early promising results for oxytocin’s effects on social cognition and behavior, more recent evidence has been mixed [15]and generally associated with small effects [20]. Researchers have long recognized the role of individual differences in the response to oxytocin treatment [1], but the focus of these differences have tended to be psychological in nature (e.g., personality), with comparatively little consideration of the impact of age [4], which is especially relevant considering *OXTR* expression fluctuations across the human lifespan. With the average age of industrialized populations on the rise, research on the needs and interests of older individuals is required where the focus should not only be on living longer, but also living better. Given the emerging research indicating the fluctuating role of the oxytocin system across the lifespan in both cognitive and somatic processes, treatments targeting the oxytocin system are a promising avenue to help address the health needs of aging populations. The neglect of oxytocin research in older populations also represents a missed opportunity, as evaluating the neurobiological mechanisms underpinning the decline of social cognition in healthy aging may help researchers better understand oxytocin’s role in social cognition difficulties in younger people with neurodevelopmental disorders. Integrating studies investigating exogenous effects of administered oxytocin and endogenous levels of oxytocin across age groups can help us better understand oxytocin’s dynamic and allostatic role across somatic and psychological processes throughout the lifespan.

We are certainly not the first to state the necessity of expanding the age diversity of samples used in oxytocin research (e.g., [11], [9]). However, the value of a lifespan approach that integrates oxytocin’s psychological and somatic effects is currently underrecognized. Pairing longitudinal and cross-sectional studies—using both exogenous administration and the measurement of endogenous levels—with genetics and neurobiology can better help triangulate evidence [16], [21], which can lead to more robust conclusions on the impact of age on oxytocin signaling and how targeting the oxytocin system might improve health and wellbeing across the lifespan.

## CRediT authorship contribution statement

**Kristin Audunsdottir:** Conceptualization, Writing – original draft. **Daniel S. Quintana:** Conceptualization, Funding acquisition, Writing – review & editing.
